# The Effects of Excess Copper on Antioxidative Enzymes, Lipid Peroxidation, Proline, Chlorophyll, and Concentration of Mn, Fe, and Cu in *Astragalus neo-mobayenii*

**DOI:** 10.1100/2012/615670

**Published:** 2012-11-20

**Authors:** P. Karimi, R. A. Khavari-Nejad, V. Niknam, F. Ghahremaninejad, F. Najafi

**Affiliations:** ^1^Faculty of Biological Sciences, Kharazmi University, Tehran 15719-14911, Iran; ^2^Department of Biology, Faculty of Science, Islamic Azad University, Science and Research Branch, Tehran 14778-93855, Iran; ^3^School of Biology and Center of Excellence in Phylogeny of Living Organisms, College of Science, University of Tehran, Tehran 14115-154, Iran

## Abstract

To probe the physiological and biochemical tolerance mechanisms in *Astragalus neo-mobayenii* Maassoumi, an endemic plant around the Cu-rich areas from the North West of Iran, the effect of different copper concentrations at toxic levels on this plant was investigated. Copper was applied in the form of copper sulfate (CuSO_4_ *·*5H_2_O) in four levels (0, 50, 100, and 150 **μ**M). We observed no visible symptoms of Cu toxicity in this plant species. During the exposure of plants to excess copper, the antioxidant defense system helped the plant to protect itself from the damage. With increasing copper concentration, superoxide dismutase (SOD), peroxidase (POD), and catalase (CAT) activities increased in leaves and roots (*P* < 0.001) compared with that of the control group. The chlorophyll amount gradually declined with increasing Cu concentrations. However, reduction in the 50 **μ**M level showed insignificant changes. Enhanced accumulation of proline content in the leaves was determined, as well as an increase of MDA content (oxidative damage biomarker) (*P* < 0.001). The results indicated that Cu contents in leaves and roots enhanced with increasing levels of Cu application. The Fe and Mn contents in both shoots and roots significantly decreased with increasing Cu concentration. Finally, the mechanisms of copper toxicity and copper tolerance in this plant were briefly discussed.

## 1. Introduction

Copper, an essential element for normal plant growth and metabolism [1, 2], plays a significant role in a number of physiological processes such as the photosynthetic and respiratory electron transport chains [3], nitrogen fixation, protein metabolism, antioxidant activity, cell wall metabolism, and hormone perception [2, 4, 5]. As a structural and catalytic component of proteins and enzymes, it is also well documented [6] and has been reported to be among the most toxic heavy metals [7]. However, when absorbed in excess quantities, Cu is highly toxic to plant growth potentially leading to physiological disorders that inhibit plant growth [8, 9]. It has been reported that excess Cu, at the cellular level, causes molecular damage to plants via the generation of reactive oxygen species (ROS) and free radicals [10]. Oxidative stress by formation of ROS and oxidation of biomolecules such as lipids, proteins, nucleic acids, carbohydrates, and almost every other organic constituent of the living cell is an important aspect of Cu toxicity [11–13]. Plant cells can be protected from ROS by enzymatic defense mechanisms like superoxide dismutase (SOD), catalase (CAT), and peroxidase (POD) and nonenzymatic defense mechanisms like free amino acids especially proline, ascorbate, and glutathione and phenolic compounds [12, 14]. Free proline is known to accumulate under heavy metal exposure and considered to be involved in stress resistance [15]. In addition, Cu toxicity is related to disturbances in the uptake and transport of other mineral elements [16]. Less is known about the effects of Cu transport and uptake on Fe, Mn, Mg, and other mineral element assimilation. The induced deficiency of mineral content under excess copper from previous investigations is also available [16–20]. *Astragalus *with nearly 3000 species is generally considered the largest genus of vascular plants. Iran is one of the largest centers of diversity for the genus. It has nearly 750 species and an endemism rate of nearly 50% [21, 22]. It was determined how some physiological and biochemical parameters and Cu, Fe, and Mn concentration in roots and shoots were changed due to excess Cu in *Astragalus* plants grown in heavy metal soils constituting the flora of Iran Northwest.

## 2. Materials and Methods

### 2.1. Seeds Germination and Growth Conditions

*Astragalus* (*A. neo-mobayenii *Maassoumi) seeds were collected from Cu-rich areas (East Azerbaijan Province, Iran) and sterilized in 1% active sodium hypochlorite solution for 5 min, carefully washed by deionized water, and germinated on damp filter paper in the dark. Six-day seedlings were transferred to appropriate light conditions and supplied with 20%, 50%, and the whole Hoagland solution for 10 days. Seedlings were then cultivated in polyethylene pots containing perlite and vermiculite, and treatments were applied after three weeks. Seedlings were grown for 30 d in a growth chamber (greenhouse) at 65% constant relative humidity, 16/8 h day/night regime under 600 *μ* mol m^−2^ s^−1^ of light intensity, and day/night temperatures 25/20°C. Plants were supplied with the Hoagland nutrient solution (pH 6.2) which contained (macronutrients in mM) 1 KH_2_PO_4_, 2 MgSO_4_·4H_2_O, 5 KNO_3_, and 5 Ca(NO_3_)_2_·4H_2_O and (micronutrients in *μ*M) 9 MnCl_2_·4H_2_O, 4.6 H_3_BO_3_, 0.8 ZnSO_4_·7H_2_O, 0.3 CuSO_4_·5H_2_O, and 0.1 H_2_MoO_4_·H_2_O. Iron was supplied as Fe-EDTA (1.8 mM). Copper in four levels (0, 50, 100, and 150 *μ*M) as CuSO_4_·5H_2_O was added to the nutrient solution. The experiment was conducted in four treatments with four replicates. 30 days after treatment, plants were harvested and used for physiological and biochemical analysis.

### 2.2. Photosynthetic Pigments' Analysis

Photosynthetic pigments (chlorophylls and carotenoids) were extracted by 80% acetone and centrifuged at 3000 g for 5 min [23]. Absorbance was determined in supernatant spectrophotometrically at 645 nm (Chl*b*), 663 nm (Chl*a*), and 470 nm (Car), and according to the Lichtenthaler and Wellburn formulae [24], pigment concentrations were calculated.

### 2.3. Enzyme Activity

The plant material (fresh weight) was homogenized on ice with 5 mL of 50 mmol sodium phosphate buffer (pH 7) including 0.5 mmol EDTA and 0.15 mol NaCl, in a mortar and pestle. The homogenate was centrifuged at 12000 g for 15 min at 4°C. The supernatant was used for enzyme assays. The activity of SOD was determined as described by Chen and Pan [25] in a 3 mL reaction mixture containing 50 mmol sodium phosphate buffer (pH 7), 10 mmol methionine, 1.17 mmol riboflavin, 56 mmol NBT, and 100 *μ*L enzyme extract spectrophotometrically at 560 nm based on the photoreduction of nitroblue tetrazolium (NBT). The blue formazan produced by NBT photoreduction was measured by an increase in absorbance at 560 nm. An SOD unit was defined as the amount of enzyme required to inhibit 50% of the NBT photoreduction. 

The activity of CAT was determined as described by Havir and McHale [26] by a decrease in absorbance of the reaction mixture at 240 nm. The activity was assayed for 1 min in a reaction solution composed of 2.9 mL potassium phosphate buffer 50 mmol (2.85 mL, pH 7.0), H_2_O_2_ 12.5 mmol (50 *μ*L), and 100 *μ*L of crude extract. The enzyme activity was calculated using the molar extinction coefficient of 36 M^−1^ cm^−1^.

Peroxidase activity was determined based on an increase in absorbance at 470 nm as described by Sakharov and Ardilla [27]. The mixture composed of 2.8 mL guaiacol (3%), 100 *μ*L H_2_O_2_ and 100 *μ*L enzyme extract. A POD unit was defined as an increase in absorbance of 1.0 per min.

### 2.4. Determination of Lipid Peroxidation

Lipid peroxidation in roots was determined using thiobarbituric acid test by measurement of malondialdehyde level [28]. Roots were homogenized in 20% trichloroaceticacid (TCA) containing 0.5% thiobarbituric acid (TBA). The extracts were centrifuged at 10000 g for 15 min after incubation in 95°C water bath for 30 min and immediately ice bath. The amount of MDA-TBA complex was calculated by its specific absorbency at 532 nm in supernatant. Nonspecific absorbency at 600 nm was also subtracted [29]. The data was obtained as nm gr^−1^ FW using the extinction coefficient of 155 mM^−1^ cm^−1^.

### 2.5. Proline Content

To estimate proline content of shoot, according to the Bates et al. [30] method, samples were homogenized in sulphosalicylic acid. The homogenate was filtered through Whatman's no. 1 filter paper. The filtrate was boiled for 1 hr after adding acetic acid and acid ninhydrin, and absorbance was taken at 520 nm wavelength. 

### 2.6. Plant Sampling and Digestion

Roots and shoot were separated and washed by double-distilled water for at least four times. The samples were oven dried at 80°C for 48 hours and then milled by mixer. Homogenate powder was weighted (150 mg) and digested in 10 mL concentrated HNO_3_ at 300°C heating plate. Cooled digests were diluted to 50 mL by double-distilled water and then filtrated by Whatman's no. 1 paper [31]. 

### 2.7. Metal Analysis

Metal contents of prepared samples were analyzed by ICP-OES spectroscopy (Varian VISTA-MPX) for manganese (Mn), cupper (Cu), and (Fe). The metal concentrations were calculated as *μ*g gr^−1^ DW.

### 2.8. Statistical Analysis

Statistical analysis were determined both based on one-way analysis of variance (ANOVA) and least significant difference (LSD) test with SPSS at significance levels of *P* < 0.001, *P* < 0.01, *P* < 0.05. 

## 3. Results and Discussion

**Figure 1:**
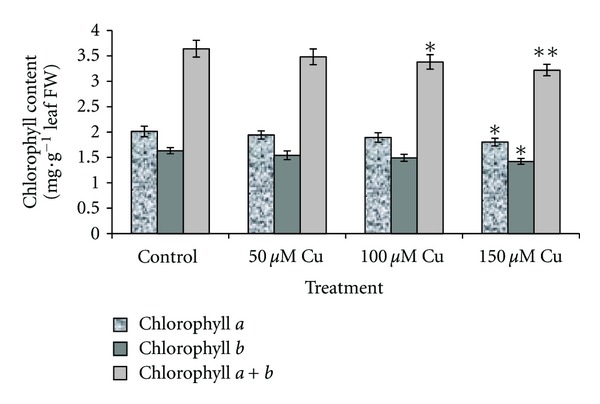
Chlorophyll *a* and chlorophyll *b* content and chlorophyll (*a* + *b*) in leaf tissues of *A. neo-mobayenii* grown in different concentrations of copper. Vertical bars represent standard error of the mean (*n* = 4). Asterisks indicate that the mean values are significantly different between treatments and control (*P < 0.05, **P < 0.01) according to LSD.

Total chlorophyll (*a* + *b*) content varied with Cu levels. With the increasing Cu concentration, the chlorophyll *a* and *b* content decreased gradually. However, reduction in the 50 and 100 *μ*M levels showed insignificant changes compared with that of the control group but showed significant change in 150 *μ*M (*P* < 0.05) (Figure 1). Reduction of chlorophyll content in plants due to excess copper was also observed by Quzounidou [32]; Rama Devi and Prasad [33]; Monni et al. [34]; Xiong et al. [35]; Singh et al. [36]. It has been proposed that Cu at toxic concentration interferes with enzymes associated with chlorophyll biosynthesis and protein composition of photosynthetic membranes [37–39]. Also, possibility of Cu-induced Fe deficiency [16] and displacing Mg required for chlorophyll biosynthesis [40] have been proposed as a damage mechanism.

**Figure 2:**
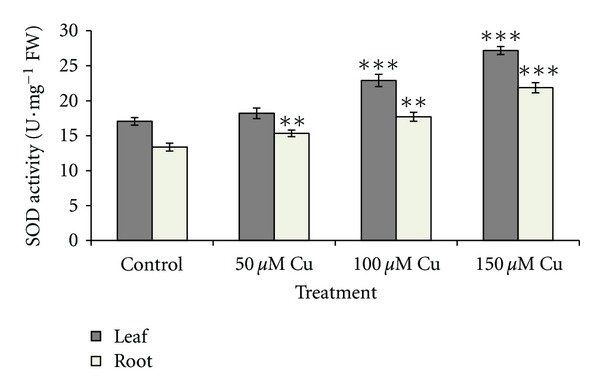
Effects of different concentrations of copper on superoxide dismutase (SOD) activity in leaves and roots of *A. neo-mobayenii*. Vertical bars represent standard error of the mean (*n* = 4). Asterisks indicate that the mean values are significantly different between treatments and control (*P < 0.05, **P < 0.01, ***P < 0.001) according to LSD.

**Figure 3:**
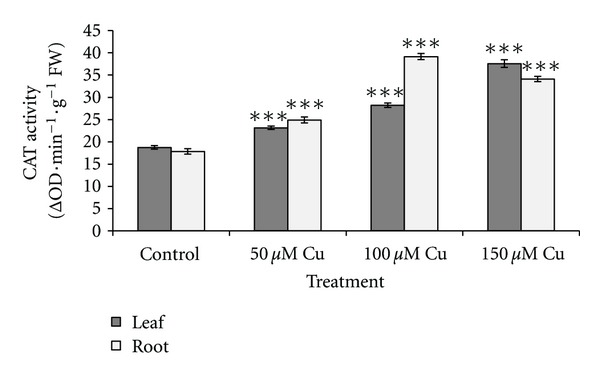
Effects of different concentrations of copper on catalase (CAT) activity in leaves and roots of *A. neo-mobayenii*. Vertical bars represent standard error of the mean (*n* = 4). Asterisks indicate that the mean values are significantly different between treatments and control (*P < 0.05, **P < 0.01, ***P < 0.001) according to LSD.

**Figure 4:**
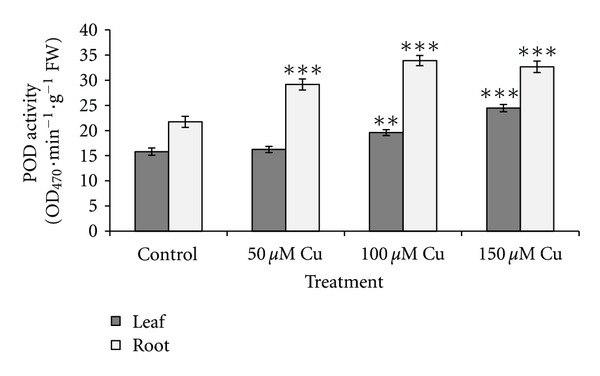
Effects of different concentrations of copper on peroxidase (POD) activity in leaves and roots of *A. neo-mobayenii*. Vertical bars represent standard error of the mean (*n* = 4). Asterisks indicate that the mean values are significantly different between treatments and control (*P < 0.05, **P < 0.01, ***P < 0.001) according to LSD.

**Figure 5:**
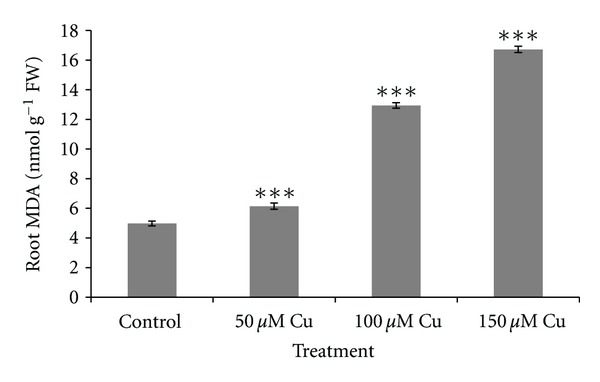
MDA levels in roots of *A. neo-mobayenii* grown in different concentrations of copper. Vertical bars represent standard error of the mean (*n* = 4). Asterisks indicate that the mean values are significantly different between treatments and control (*P < 0.05, **P < 0.01, ***P < 0.001) according to LSD.

Figure 2 shows the changes of SOD activity in leaves and roots. No significant changes in SOD activity were observed in the leaves under 50 *μ*M Cu concentration, while the activities showed significant increases (*P* < 0.001) under higher level of Cu concentration. Significant increases in root SOD activities under all treatments were observed (*P* < 0.05 or *P* < 0.001). As Cu concentration increased, the root CAT activity increased significantly (*P* < 0.01). The same result was observed in leaves as shown in Figure 3.  Figure 4 shows increased POD activity in both leaves and roots concomitantly with increased Cu level. The increase in POD activity in both was significant (*P* < 0.001). The result of lipid peroxidation in root in the control and treatment groups is shown in Figure 5. MDA level in roots significantly increased with the increase of Cu concentration (*P* < 0.001). Our studied plant was endemic around the Cu-rich area and had adapted to contaminated soils by developing tolerance mechanisms to this metal stress. Many studies reported that internal protective responses to excess copper can vary among plant species and among different tissues [41]. It is well known that when copper is in excess, it catalyzes the formation of ROS and particularly, the highly toxic hydroxyl radicals from Haber-Weiss reaction [42], leading to an increase in MDA as biomarkers of oxidative damages. Hence, in response to the presence of excess Cu, plants increased the antioxidant responses due to increased generation of ROS. Accordingly, it was observed an excess Cu in plants inducing defense genes responsible for antioxidant enzymes, including SOD, POD, and CAT, which contribute to the removal of ROS [43–46]. SOD catalyzes the dismutation of superoxide into oxygen and hydrogen peroxide. The enhanced activity of catalase demonstrated that any hydrogen peroxide formed as a result of SOD activity was consumed by catalase and/or peroxidase. This indicated that these enzymes were known as a mediator of oxidative damage and might be sufficient to protect biomolecules of some parts of plants against ROS attack [13].

**Figure 6:**
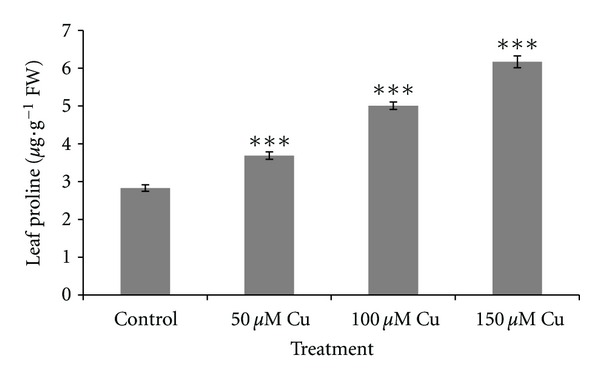
Proline contents in leaves of *A. neo-mobayenii* grown in different concentrations of copper. Vertical bars represent standard error of the mean (*n* = 4). Asterisks indicate that the mean values are significantly different between treatments and control (*P < 0.05, **P < 0.01, ***P < 0.001) according to LSD.

Figure 6 shows Cu-induced proline accumulation in shoots. The proline content increased substantially with increasing Cu concentrations (*P* < 0.001). This may be because synthesis of proline is considered to be one of the first metabolic responses to stress and acts osmoregulator, stabilizer of protein synthesis, a metal chelator, and a hydroxyl radical scavenger [47–49].

The Cu content in shoots and roots increased significantly with an increase in the level of applied Cu. The accumulations in shoots were higher than that of roots in all treatments. Fe content in both shoots and roots reduced with increasing Cu concentration in the medium. However, a slight increase was observed in the lower level of applied Cu. The Mn content decreased insignificantly at higher levels of applied Cu. In roots increased levels of Mn were observed (Table 1). The results are in close conformity with the findings that an elevated copper application resulted in an increase in plant Cu content [8, 50–52]. In high concentration of copper application, the copper levels in leaves were above the threshold for copper toxicity [4]. On the other hand, normal growth of studied plants without any visible symptoms of Cu toxicity implied that this plant was tolerant to toxic levels of Cu. In addition, translocation of copper to the shoots was suggested as a strategy to explain the copper tolerance mechanism developed by plant in order to reduce copper stress. Thus according to the present study, this plant could be suitable for phytoextraction [53, 54].

Interference of Cu and Cd with the root uptake of mineral nutrients has been observed [55, 56]. Moreover, antagonistic effects of Cu and Fe have been suggested by many workers and often occur in plants grown under Cu toxicity [17, 57–59]. Also, competition of copper with Mn for transport sites in plasmalemma has been reported [60, 61]. In this study reduction of Mn with increasing levels of copper was observed. However, Mn contents in leaves did not drop below the critical deficiency range [4].

## Figures and Tables

**Table 1:** Effects of excess copper on Cu, Mn, and Mg contents of the shoots and roots of *A. neo-mobayenii*.

Cu (*μ*M)	Shoot			Root		
	Cu (*μ*g/g DW)	Mn (*μ*g/g DW)	Fe (mg/g DW)	Cu (*μ*g/g DW)	Mn (*μ*g/g DW)	Fe (mg/g DW)
Control	12.32	42.9	147	8.14	24.67	93
50	23.69	42.72^NS^	146	14.73	26.12	85
100	31.92	39.14	119	18.12	26.89	67
150	44.58	36.12	106	24.29	25.09	53

Each value is the mean of the four replications.

All the values are significant at *P* <0.01.

NS: nonsignificant.
